# Impact of genetically predicted characterization of mitochondrial DNA quantity and quality on osteoarthritis

**DOI:** 10.3389/fgene.2023.1130411

**Published:** 2023-02-24

**Authors:** Houpu Liu, Bingyue Cai, Ruicheng Gong, Ye Yang, Jing Wang, Dan Zhou, Min Yu, Yingjun Li

**Affiliations:** ^1^ Department of Epidemiology and Health Statistics, School of Public Health, Hangzhou Medical College, Hangzhou, China; ^2^ Starbody Clinic, Hangzhou, China; ^3^ Department of Big Data in Health Science, School of Public Health, Zhejiang University School of Medicine, Hangzhou, China; ^4^ Vanderbit Genetics Institute, Vanderbilt University Medical Center, Nashville, TN, United States; ^5^ Zhejiang Provincial Center for Disease Control and Prevention, Hangzhou, China

**Keywords:** mitochondrial heteroplasmy, mitochondrial abundance, osteoarthritis, mendelian randomization, knee OA (KOA)

## Abstract

**Background:** Existing studies have indicated that mitochondrial dysfunction may contribute to osteoarthritis (OA) development. However, the causal association between mitochondrial DNA (mtDNA) characterization and OA has not been extensively explored.

**Methods:** Two-sample Mendelian randomization was performed to calculate the impact of mitochondrial genomic variations on overall OA as well as site-specific OA, with multiple analytical methods inverse variance weighted (IVW), weighted median (WM), MR-Egger and MR-robust adjusted profile score (MR-RAPS).

**Results:** Genetically determined mitochondrial heteroplasmy (MtHz) and mtDNA abundance were not causally associated with overall OA. In site-specific OA analyses, the causal effect of mtDNA abundance on other OA sites, including hip, knee, thumb, hand, and finger, had not been discovered. There was a suggestively protective effect of MtHz on knee OA IVW OR = 0.632, 95% CI: 0.425–0.939, *p*-value = 0.023. No causal association between MtHz and other different OA phenotypes was found.

**Conclusion:** MtHz shows potential to be a novel therapeutic target and biomarker on knee OA development. However, the variation of mtDNA abundance was measured from leukocyte in blood and the levels of MtHz were from saliva samples rather than cartilage or synovial tissues. Genotyping samples from synovial and cartilage can be a focus to further exploration.

## 1 Introduction

Osteoarthritis (OA) is a common joint disease with degenerative changes in articular cartilage and remains major impact on public health worldwide (Nelson, 2018). According to the estimation of the Global Burden of Disease Study (GBD), nearly 300 million individuals throughout the world are suffering from OA, and the number is still increasing. OA is one of the leading causes of pain, disability, and great socioeconomic burden in developed countries (Glyn-Jones et al., 2015). However, the mechanism of OA remains unclear. Identifying risk factors such as a disorder-related biomarker is essential to understand OA pathogenesis, decrease the incidence of the disease and develop efficiency prevention strategies.

Mitochondria are complex multifunctional organelles involved in various cell functions, e.g., heat regulation, calcium homeostasis, reactive oxygen species (ROS) production, and proinflammatory cytokines production, the damage of which may affect chondrocyte health at an extent (Blanco et al., 2011; Fernandez-Moreno et al., 2017; Blanco et al., 2018). They have their own genome (mitochondrial DNA, mtDNA) which contain 37 genes and encode 13 proteins, 22 transfer RNAs (tRNAs) and two ribosomal RNAs (rRNAs) (Anderson et al., 1981). As multicopy genome, sequence mutation and copy number variation of mtDNA are prevalent in population (McArdle et al., 2013; McGuire, 2019). As one of the mtDNA quantity characteristics, mtDNA abundance is recognized as a rough estimation for the number of mitochondria (Castellani et al., 2020). Previous studies provided supportive evidence for extensive pathogenicity of mtDNA abundance (Frahm et al., 2005; Hu et al., 2016). As one of the mtDNA quality characteristics, mitochondrial herteroplasmy (MtHz), where mtDNA with distinct sequences coexist, has been found in a large spectrum of human disease, including classical mitochondrial diseases and complex disorders (Ye et al., 2014). MtDNA abundance and MtHz can integrate many aspects of mitochondrial function and serve as promising biomarkers in probing interactions between mitochondria and disorders (Castellani et al., 2020; Tian et al., 2021).

In a recent review, OA was recognized as a potential mitochondrial disease considering the impact of mitochondrial dysfunction on cartilage degradation (Fernandez-Moreno et al., 2022). However, the causality between mitochondria and OA has rarely been investigated. Therefore, the goal of our study was to probe causal effects of characterization of mtDNA quantity and quality on OA development which is crucial to understand the role of mitochondria in OA etiology. A two-sample Mendelian randomization (MR) analysis was performed to investigate potential causal associations between mtDNA abundance and MtHz with OA using summary statistics from large-scale genome-wide association study (GWAS). MR-Steiger test was applied to ascertain whether variation of mtDNA characterization is a cause or a consequence of OA development.

## 2 Materials and methods

### 2.1 Study design overview

**FIGURE 1:**
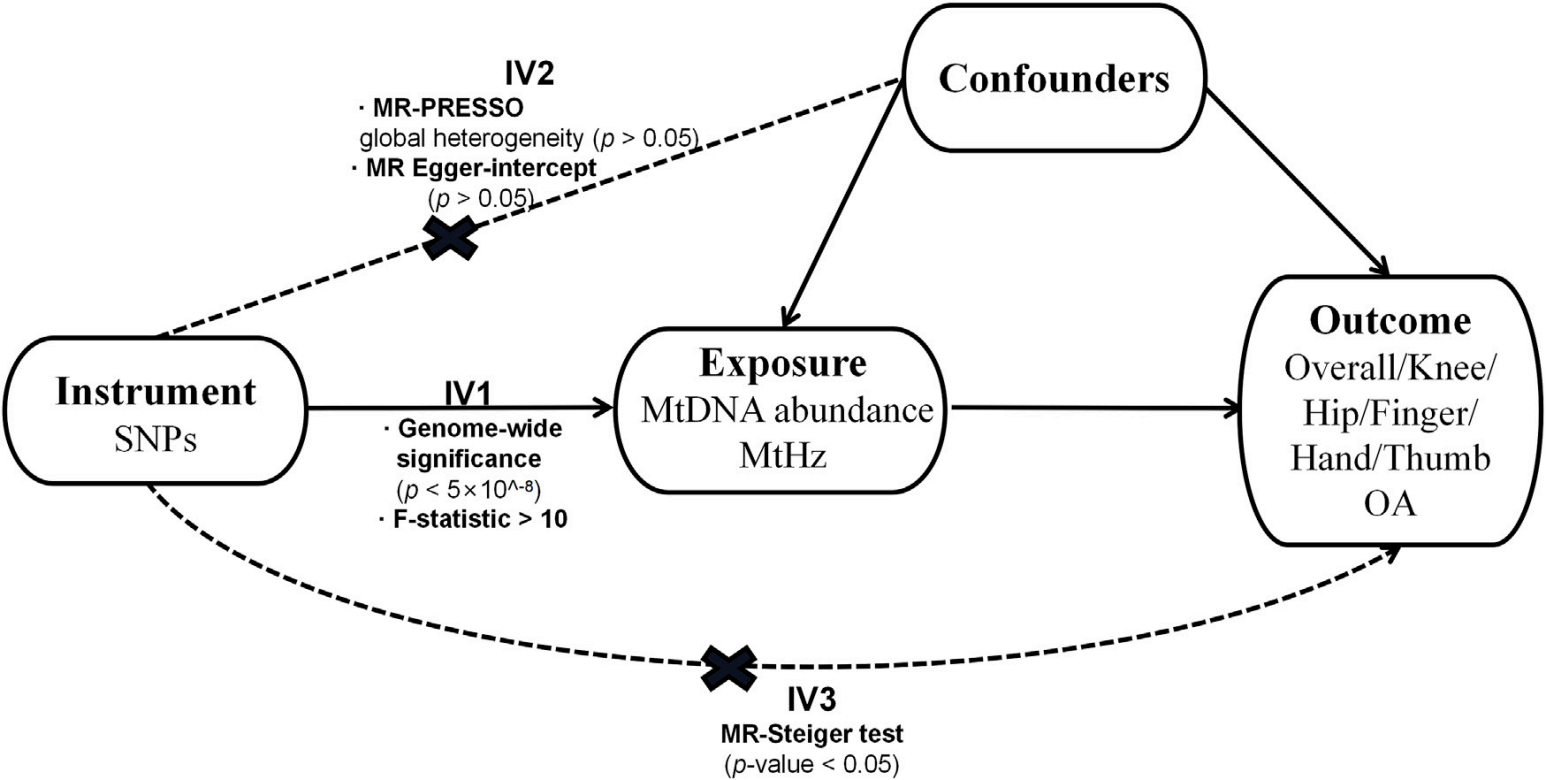
The assumptions of MR and how we tested these assumptions in our analyses.

The design of our research is displayed in Figure 1. We adopted a two-sample MR design to compute the causal effect of the characterization of mtDNA quantity and quality on overall OA and site-specific OA separately (Boer et al., 2021). Genetic association estimates for mtDNA abundance were derived from the United Kingdom biobank study (N = 291,950) (Hagg et al., 2021). Single Nucleotide Polymorphisms (SNPs) associated with MtHz were obtained from the 23andME research program (N = 982,072), a personal genomics and biotechnology company (Nandakumar et al., 2021). The participants included in both GWASs were of European ancestry.

### 2.2 Selection of genetic instruments

SNPs serving as instrumental variables (IVs) for MtHz and mtDNA abundance, all reach genome-wide significance (*p* < 5 × 10–8) (Hagg et al., 2021; Nandakumar et al., 2021). Twenty SNPs were related to MtHz and accounted for 32% observed SNP-heritability, 64 SNPs were included to estimate the genetic liability of mtDNA abundance and explained approximately 8.3% SNP-heritability. We performed linkage disequilibrium (LD) pruning in PLINK with 1,000 Genomes Europeans as the reference panel to ascertain whether these genetic variants are independent of each other (*r* ^2^ < 0.001) (Burgess et al., 2017). After LD test, five genetic variants linked to MtHz were removed, and 17 IVs related to mtDNA abundance were excluded. These SNPs were then matched in summary GWASs of OA in subsequent analysis and those not available in the outcome GWAS were removed or replaced by the proxy SNPs. F-statistic was calculated to filter the weak instruments. The threshold of F-statistic that was sufficient for identifying causal effect was 10 (Burgess et al., 2011). The selected genetic variants are demonstrated in Supplementary Table S1, S2.

### 2.3 Osteoarthritis and data sources

Summary statistics data on overall OA and its specific sites, comprising knee, hip, spine, thumb, and hand OA, were obtained from the latest publicly available GWAS of OA. This GWAS covered nine populations with up to 826,690 participants (177,517 OA patients) (Boer et al., 2021). The definition of OA satisfied the criteria of Genetics of Osteoarthritis (GO) including self-reported status, hospital diagnosed, ICD10 codes or radiographic as defined by the TREAT-OA consortium (Boer et al., 2021). All studies contributing data to our analyses were approved by the relevant ethics committees, and all study participants in these studies provided written, informed consent.

### 2.4 Statistical analysis

**FIGURE 2:**
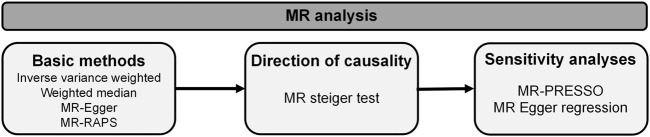
Flowchart of Mendelian randomization framework in this study.

Two-sample MR analysis was executed in R software (R version 4.3.0) with the TwoSampleMR (version 0.5.6), MR-PRESSO (version 1.0) and mr. raps (version 0.2) R packages (Hemani et al., 2018; Verbanck et al., 2018). The methods applied in our research are presented in Figure 2. MR-steiger test was applied to infer causal direction between traits under investigation (Hemani et al., 2017). Four methodologies including inverse variance weighted (IVW), weighted median (WM), MR-Egger and MR-robust adjusted profile score (MR-RAPS) were employed to estimate causality between mtDNA abundance and MtHz and OA. IVW was taken as a primary MR analysis method in our research, which is based on the hypothesis that all selected genetic instruments are valid and give an overall causal estimate strengthening causal inference (Lawlor et al., 2008; Burgess et al., 2013). MR-Egger regression takes presence of directional pleiotropy into consideration and measures horizontal pleiotropy with regression intercept, whereas the results of this method are susceptible to outlying genetic variants (Bowden et al., 2015). Weighted Median based on hypothesis that at least 50% of the variants are valid, improves power of causal effect detection but reduces precision (Bowden et al., 2016). MR-RAPS applies robust adjusted profile scores to correct for pleiotropy and makes our results more reliable (Hartwig et al., 2017).

Several sensitivity analyses were applied to detect and correct for heterogeneity and pleiotropy. MR-PRESSO was conducted to detect the existence of horizontal pleiotropy and correct the causal estimate affected by possible pleiotropic outliers (Verbanck et al., 2018). Corresponding *p*-values were derived based on 1,000 simulations (Verbanck et al., 2018). And the estimation of MR Egger regression intercept was also employed to reflect presence of pleiotropy (*p* < 0.05) (Burgess and Thompson, 2017). IVW method was utilized to investigate heterogeneity. The level of heterogeneity was quantified by Cochran Q statistics (Carnegie et al., 2020). Leave-one-out sensitivity analysis was performed to identify possibly influential SNPs, which repeated MR analysis with each SNP excluded in turn (Carnegie et al., 2020). As five separate outcomes were tested in our study, main results had statistical significance at *p*-value<0.01 (0.05/5) after Bonferroni correction.

## 3 Results

### 3.1 Causal effect of mitochondrial heteroplasmy on OA

Fifteen LD-independent genetic variants were taken for repeated MR analysis (Supplementary Table S1). We extracted data of above-mentioned SNPs from summary GWAS of outcome traits (overall OA and specific-site OA) and one SNP (rs2286639) was removed in all outcomes except finger OA due to the effect of non-concordant alleles (e.g., A/G vs A/C). In finger OA, two SNP (rs2286639, rs3702096) were excluded for absence of proxy SNPs on the online platform SNiPA (https://snipa.helmholtzmuenchen.de/snipa3/). The mean F-statistic was 36.357 which was above the threshold F value of 10. In total, there were 14 IVs for overall, knee, hip, thumb, and hand OA and 13 IVs for finger OA.

Results of the casual association between MtHz and OA are summarized in Table 1. The primary IVW analysis provided no evidence for the casual association between MtHz and overall OA [odds ratio (OR) = 0.852, 95% confidence interval (CI): 0.664–1.093, p= 0.208]. The results of WM method, MR-RAPS and MR-Egger were consistent with the result of IVW. Both MR-Egger and MR-PRESSO reported no existence of horizontal pleiotropy (Egger-intercept: *p* > 0.05; MR-PRESSO global heterogeneity: *p* > 0.05). Cochran’s Q test did not detect heterogeneity in overall OA (p= 0.495 > 0.05).

**TABLE 1:** MR results of causal effect of mitochondrial heteroplasmy on OA and its other phenotypes.

Outcome traits	MR methods	Mitochondrial heteroplasmy					
		OR (95%CI)	SE	MR *p*-value	Heterogeneity test	Pleiotropy test	MR-stegier test
					Cochran’s Q (*p*)	*p* intercept	
Overall OA	MR-Egger	0.922 (0.619,1.373)	0.203	0.697	12.407 (0.495)	0.625	Direction: TRUE*p*-value < 0.0001
	Inverse variance weighted	0.852 (0.664,1.093)	0.127	0.208			
	MR-RAPS	0.850 (0.662,1.093)	0.128	0.206			
	Weighted median	0.867 (0.640,1.174)	0.155	0.355			
	MRPRESSO	0.851 (0.670,1.082)	0.123	0.209			
Knee OA	MR-Egger	0.667 (0.358,1.243)	0.318	0.226	11.272 (0.588)	0.831	Direction: TRUE*p*-value < 0.0001
	Inverse variance weighted	**0.632 (0.425,0.939)**	0.202	**0.023**			
	MR-RAPS	0.630 (0.422,0.939)	0.204	**0.023**			
	Weighted median	0.637 (0.402,1.009)	0.235	0.055			
	MRPRESSO	**0.631 (0.442,0.903)**	0.182	**0.024**			
Hip OA	MR-Egger	0.708 (0.320,1.568)	0.406	0.412	10.208 (0.676)	0.897	Direction: TRUE*p*-value < 0.0001
	Inverse variance weighted	0.738 (0.447,1.221)	0.257	0.237			
	MR-RAPS	0.734 (0.442,1.220)	0.259	0.234			
	Weighted median	0.711 (0.391,1.293)	0.310	0.272			
	MRPRESSO	0.736 (0.466,1.161)	0.233	0.208			
Thumb OA	MR-Egger	0.708 (0.320,1.568)	0.406	0.412	10.161 (0.681)	0.288	Direction: TRUE*p*-value < 0.0001
	Inverse variance weighted	1.270 (0.506,3.188)	0.469	0.611			
	MR-RAPS	1.270 (0.502,3.216)	0.474	0.615			
	Weighted median	0.711 (0.391,1.293)	0.305	0.264			
	MRPRESSO	1.267 (0.576,2.791)	0.403	0.566			
Hand OA	MR-Egger	0.967 (0.333,2.803)	0.543	0.951	7.958 (0.846)	0.432	Direction: TRUE*p*-value < 0.0001
	Inverse variance weighted	1.357 (0.687,2.681)	0.347	0.379			
	MR-RAPS	1.350 (0.678,2.686)	0.351	0.392			
	Weighted median	1.132 (0.503,2.546)	0.413	0.764			
	MRPRESSO	1.348 (0.769,2.362)	0.286	0.315			
Finger OA	MR-Egger	0.139 (0.010,1.909)	1.336	0.168	8.157 (0.773)	0.251	Direction: TRUE*p*-value < 0.0001
	Inverse variance weighted	0.483 (0.090,2.588)	0.857	0.395			
	MR-RAPS	0.481 (0.088,2.621)	0.865	0.398			
	Weighted median	0.364 (0.049,2.688)	1.021	0.322			
	MRPRESSO	0.483 (0.121,1.927)	0.707	0.323			

MR, mendelian randomization; SNP, single nucleotide polymorphism; OA, osteoarthritis; OR, odds ratio; CI, confidence interval; SE, standard error (the standard error is an estimate of the standard deviation (SD) of the coefficient); *p*, *p*-value.

In site-specific OA analyses, the nominally significant results of IVW analysis suggested that MtHz was a potentially protective factor for knee OA (OR = 0.632, 95% CI: 0.425–0.939, p= 0.023, Table 1). MR-RAPS showed similar results with IVW analysis (OR = 0.629, 95% CI: 0.422–0.939, p= 0.023, Table 1). However, no relationship between MtHz and other OA sites, including hip OA (IVW OR = 0.738, 95% CI: 0.447–1.221, p= 0.237), hand OA (IVW OR = 1.357, 95% CI: 0.687–2.681, p= 0.379), thumb OA (IVW OR = 1.270, 95% CI: 0.506–3.188, p= 0.611) or finger OA (IVW OR = 0.483, 95% CI: 0.090–2.588, p= 0.395) was observed in IVW model (Table 1). MR-PRESSO was performed to test for horizontal pleiotropy, and no outliers was identified. The results of MR-Egger intercept were consistent with the MR-PRESSO results (intercept *p* > 0.05). The heterogeneity was tested by Cochran’s Q test and MR-PRESSO global heterogeneity test, providing no evidence about the existence of heterogeneity. The MR-Steiger results supported that these SNPs were more predictive of the exposure than of the outcome (*p* < 0.05, Supplementary Table S3). The results of leave-one-out sensitivity analysis and forest plots demonstrated that our study in genetically prediction was robust (Supplementary Figure S1–S3).

### 3.2 Causal effect of mitochondrial abundance on OA

After LD test, 47 independent genetic variants were chosen for two-sample MR analysis. Several genetic variants could not be matched in summary statistic GWASs of knee (rs35734242), finger (rs1065853), and hand OA (rs12451555). We searched their proxy SNPs on SNiPA and those whose proxy SNPs were absent on SNiPA were excluded. The mean F-statistic for IVs of mtDNA abundance was 93.380, which was above the threshold 10 (Supplementary Table S2). Finally, the SNPS were selected as IVs for thumb, hip, overall OA was 47, and 46 SNPS for hand, finger, knee. Regarding overall OA, MR-PRESSO identified two outliers s (rs59488041, rs12924138) and therefore removed them for repeated MR analysis. In the presence of heterogeneity (Cochran’s Q: p= 0.033) and absence of pleiotropy (Egger-intercept: p= 0.687 > 0.05), the results of WM analysis on overall OA were nominally significant (OR = 1.108, 95% CI: 1.001–1,226, p= 0.048, Table 2). IVW method in random effects model was utilized to correct results potentially impacted by heterogeneity, and the results suggested that genetically elevated mtDNA abundance was not casually associated with overall OA (OR = 1.040, 95% CI: 0.963–1.125, p= 0.318). MR-RAPS analysis agreed with the results of IVW analysis (OR = 1.041, 95% CI: 0.974–1.112, p= 0.228, Table 2).

**TABLE 2:** MR results of causal effect of mtDNA abundance on OA and its other phenotypes.

Outcome traits	MR methods	MtDNA abundance					
		OR (95%CI)	SE	MR *p*-value	Heterogeneity test	Pleiotropy test	MR-stegier test
					Cochran’s Q (*p*)	*p* intercept	
Overall OA	MR-Egger	1.091 (0.856,1.391)	0.124	0.485	**62.748 (0.033)**	0.687	Direction: TRUE
	Inverse variance weighted	1.040 (0.963,1.125)	0.040	0.318			
	MR-RAPS	1.041 (0.974,1.112)	0.034	0.229			*p*-value < 0.0001
	Weighted median	**1.108 (1.001,1,226)**	0.052	**0.048**			
	MRPRESSO	1.040 (0.963,1.125)	0.040	0.323			
Knee OA	MR-Egger	0.823 (0.562,1.206)	0.195	0.324	65.050 (0.027)	0.225	Direction: TRUE
	Inverse variance weighted	1.034 (0.915,1.168)	0.062	0.592			
	MR-RAPS	1.035 (0.934,1.145)	0.052	0.516			*p*-value < 0.0001
	Weighted median	1.056 (0.903,1.236)	0.080	0.494			
	MRPRESSO	1.034 (0.915,1.168)	0.062	0.594			
Hip OA	MR-Egger	**0.616 (0.399,0.950)**	0.221	**0.034**	60.396 (0.062)	**0.027**	Direction: TRUE
	Inverse variance weighted	0.992 (0.857,1.152)	0.076	0.931			
	MR-RAPS	0.993 (0.873,1.130)	0.066	0.919			*p*-value < 0.0001
	Weighted median	0.921 (0.763,1.113)	0.096	0.397			
	MRPRESSO	0.993 (0.857,1.152)	0.076	0.931			
Thumb OA	MR-Egger	0.819 (0.364,1.844)	0.414	0.633	61.709 (0.061)	0.392	Direction: TRUE
	Inverse variance weighted	1.148 (0.880,1.499)	0.136	0.309			
	MR-RAPS	1.151 (0.912,1.454)	0.119	0.235			*p*-value <0.0001
	Weighted median	1.135 (0.790,1.631)	0.185	0.494			
	MRPRESSO	1.148 (0.880,1.499)	0.136	0.314			
Hand OA	MR-Egger	1.028 (0.601,1.759)	0.274	0.920	40.089 (0.680)	0.663	Direction: TRUE
	Inverse variance weighted	1.152 (0.969,1.369)	0.088	0.108			
	MR-RAPS	1.154 (0.967,1.376)	0.090	0.109			*p*-value < 0.0001
	Weighted median	1.144 (0.887,1.476)	0.130	0.299			
	MRPRESSO	1.152 (0.979,1.356)	0.083	0.096			
Finger OA	MR-Egger	0.876 (0.223,3.442)	0.698	0.850	51.688 (0.229)	0.837	Direction: TRUE
	Inverse variance weighted	1.004 (0.647,1.556)	0.224	0.987			
	MR-RAPS	1.003 (0.662,1.520)	0.212	0.986			*p*-value < 0.0001
	Weighted median	1.193 (0.641,2.221)	0.317	0.577			
	MRPRESSO	1.004 (0.647,1.556)	0.224	0.987			

MR, mendelian randomization; SNP, single nucleotide polymorphism; OA, osteoarthritis; mtDNA, mitochondrial DNA; OR, odds ratio; CI, confidence interval; SE, standard error (the standard error is an estimate of the standard deviation (SD) of the coefficient); *p*, *p*-value.

With respect to site-specific OA, we observed that genetically determined MtHz was not causally associated with knee OA (OR = 1.034, 95% CI: 0.915–1.168, p= 0.592), thumb OA (OR = 1.148, 95% CI: 0.880–1.499, p= 0.309), hand OA (OR = 1.152, 95% CI: 0.969–1.369, *p* = 0.108) and finger OA (OR = 1.004, 95% CI: 0.647–1.556, p= 0.987) in IVW model. The results are presented in Table 2. Both MR-Egger and MR-PRESSO reported no existence of horizontal pleiotropy (Egger-intercept: *p* > 0.05; MR-PRESSO global heterogeneity: *p* > 0.05). Cochran’s Q test did not detect heterogeneity in above-mentioned outcome traits (*p* > 0.05). For the analysis of hip OA, rs16978036 were excluded from MR-PRESSO analysis. In presence of horizontal pleiotropy (p= 0.027) and absence of heterogeneity (p= 0.062), the nominally significant result of MR-Egger method was found (OR = 0.616, 95% CI: 0.399–0.950, p= 0.034). However, no evidence about causal relationship between the exposure and hip OA was observed with IVW method and MR-RAPS method (IVW: OR = 0.992, 95% CI: 0.857–1.152, p= 0.931; MR-RAPS: OR = 0.993, 95% CI: 0.873–1.130, p= 0.919) (Table 2). The results of MR-Stegier test were in Supplementary Table S3.

Scatter plots, forest plots and leave-on-out sensitivity analysis plots were displayed in Supplementary Figure S4–6. The results of leave-one-out analysis implicated that the selected genetic variants potentially impacted the pooled results, which suggested that careful interpretations for the results was crucial.

## 4 Discussion

The causal roles of MtHz and mtDNA abundance in OA pathogenesis were poorly studied in previous works. To our best knowledge, this is the first MR study to evaluate the causal association between mitochondrial genome traits and OA. Four MR methods were employed to estimate the causal association between mtDNA characterization and OA. The results of MR steiger test verified the causal direction of our research (MtHz and mtDNA abundance were exposure and OA were outcome). No effect of mtDNA abundance and MtHz on overall OA was detected. In subgroup analysis, a suggestively protective role of MtHz on knee OA was observed, but not on other sites. And we did not find that mtDNA abundance was causally associated with any site-specific OA.

Although mitochondria is widely recognized as an important factor of OA development (Blanco et al., 2011), the role of MtHz has not been thoroughly investigated (Suliman and Piantadosi, 2016). Indeed, heteroplasmic mutations in mtDNA are often pathogenetic (Ye et al., 2014). An animal study has indicated that the state of heteroplasmy itself was deleterious when the two mtDNA sequences contain no pathogenic variants (Sharpley et al., 2012). However, there is potentially a particular level threshold for MtHz (McCormick et al., 2020). For instance, when the A3243G mutation in mitochondrial DNA is present in more than 10%, patients can manifest Type 2 diabetes (Wallace, 2005). And low-frequency mtDNA variants (0.2%–2% heteroplasmy) are extensively presented in healthy subjects (Payne et al., 2013). Furthermore, MtHz can be beneficial in health promotion as an intermediate state in emergence of novel mtDNA haplogroups (Wallace, 2016). In previous study, MtHz was found to be significantly relevant to several haplogroups (haplogroup H, J, K, T, U and X) with different characterizations among haplogroups (Nandakumar et al., 2021). Mitochondrial haplogroup J has been extensively found to mediate the development of OA (5). A Spanish cohort-study ( $n_{case}$ = 457; $n_{control}$ = 262) had reported that haplogroup J was associated with a decreased risk of knee OA (OR = 0.460, 95% CI: 0.282–0.748, p= 0.002) (Rego-Perez et al., 2008). Furthermore, a meta-analysis in European cohorts also suggested that haplogroup J was associated with a lower risk of knee OA (HR = 0.702, 95% CI: 0.541–0.912, p= 0.008) (Fernandez-Moreno et al., 2017). However, the association between mitochondrial DNA variants and OA has not been verified in a large sample observational study (Hudson et al., 2013). The study has only explored the causal relationship in terms of single mutation, but we take MtHz (variation at a whole mtDNA level) as an exposure which contribute to understand the causal role of mitochondria in OA development. In terms of biological mechanism, MtHz has been reported a regulation role of metabolic and epigenomic changes. Kopinski PK et al. had found the levels of mitochondrially drove acetyl-CoA decreased at high heteroplasmy (Kopinski et al., 2019). And the reduction of acetyl-CoA levels might influence histone acetylation and activate AMP-activated protein kinase (AMPK) to protect from OA development and progression (Chen et al., 2018). Besides, MtHz could play a role in other processes involved in OA pathogenesis to reduce the risk of OA, including regulating energy production, maintaining mitochondrial proteostasis, suppressing matrix metalloproteinase expression, reducing ROS generation, and promoting mitophagy (Blanco et al., 2018). In conjunction with prior observational findings and mechanism studies, our epidemiologic and genetic findings provided supportive evidence that MtHz may have therapeutic value in knee OA and can be regarded as a candidate biomarker after precisely identifying the relationship between MtHz and each haplogroup (Blanco et al., 2018).

However, the reason that MtHz showed diverse effects on different sites of OA remains unclear. So far, we did not find relevant research that investigated the relationship between MtHz and hand, finger, and thumb OA. A case-control study suggested that mitochondrial haplogroups was associated with hip OA (Rego et al., 2010) (OR = 0.661, 95% CI: 0.440–0.993, p= 0.045), however, with no evidence on the causality. In addition, the data sources and sample size used in MR analysis varied in site-specific OA, which mainly included more knee OA cases and less other skeletal joints cases. More MR studies and additional GWAS covering more other site-specific OAs are needed to determine a role of MtHz in the risk of different OA sites.

MtDNA abundance has been regarded as a potential biomarker of mitochondrial function and plays a role in several human diseases (Castellani et al., 2020; Clyde, 2022). Existing literature has suggested that mtDNA abundance may involve in production of inflammatory mediators and regulation of immune function that can influence OA development (Blanco et al., 2018; Zhan et al., 2020). In addition, mtDNA abundance is also associated with sex, advanced age, and elevated BMI, which are also risk factors for OA (16). However, there are few studies to estimate the effects of mtDNA abundance on OA. Only two case-control studies in Asian population were found that reported an association between mtDNA copy number and OA, but their findings were inconsistent (Fang et al., 2014; Zhan et al., 2020). One in Thailand ( $n_{case}$ = 204; $n_{control}$ = 169) had found mtDNA abundance in the OA group was significantly lower than that in the control group (*p* < 0.0001), whereas the results were not adjusted by sex and age (Zhan et al., 2020). Another case-control study carried out in southern Chinese ( $n_{case}$ = 187; $n_{control}$ = 420) observed a general increase of mtDNA abundance in OA patients (p= 0.019), but obesity was not adjusted in the analysis (Fang et al., 2014). These findings from case-control studies could not determine causal relationships between mtDNA abundance and OA, and the potential confounding variables were not comprehensively considered. Our MR analysis overcame these shortcomings and suggested that genetically determined mtDNA abundance was unrelated to OA. Furthermore, previous study has implicated that the relative contribution of mtDNA abundance might differ between different ethnic groups (Ruiz-Pesini et al., 2004). Considering that our studies have been carried out in European population, the comparisons with results from other ethnic groups such as Asian ancestry require careful considerations.

Our research applies MR methods to investigate causal relationships between mitochondrial genome characterization and OA. However, there are some limitations in our analysis. Firstly, we did not estimate causal effects of mitochondrial genome traits on OA stratified by gender. Mitochondrial genome are maternally inherited, and females may have lower MtHz than males from the perspective of mitochondrial inheritance (Nandakumar et al., 2021). Therefore, the effects of MtHz and mtDNA abundance on OA may differ in gender. Secondly, only participants of European descent are included in the study, but the impact of specific mtDNA variants on diseases could vary in different ethnic groups (Ruiz-Pesini et al., 2004). Additional MR studies on other ethnic groups are needed to probe a causal association between mtDNA characterization and OA. Thirdly, the genetic instruments for mtDNA abundance explain a relatively small amount of phenotypic variance (8.3%), IVs that can account for more variance of mtDNA abundance are warranted to draw robust conclusions. Besides, considering the Bonferroni correction of multiple independent tests, our findings about the association between MtHz and knee OA are deemed suggestive evidence of possible associations (0.01 < *p*< 0.05). Furthermore, the variation of mtDNA abundance and the levels of MtHz varied in different tissues and cell types. And in original GWASs, mtDNA abundance was measured from leukocyte in blood and MtHz from saliva samples rather than cartilage or synovial tissues. Considering that OA is mainly characterized by progressive loss of cartilage and synovial hyperplasia, the application of data from blood samples and saliva samples would limit the explanation ability of our study to a certain extent (Sellam and Berenbaum, 2010). Observational epidemiology studies exploring an association between concrete levels of MtHz and a risk of OA are needed to improve causal inference.

## 5 Conclusion

In conclusion, our MR analyses elucidated that MtHz is a suggestively protective factor of knee OA, implying that MtHz could be a genetically prediction factor and a therapeutic target in the development of knee OA. No causal association was found between mtDNA abundance and OA. Additional MR analyses are warranted to probe causal relationships between mitochondrial genome traits and OA stratified by gender. Moreover, GWAS covering more than one ethnic population are needed to detect the effect of characterization of mtDNA quantity and quality in different ethnic groups.

## Data Availability

The original contributions presented in the study are included in the article/Supplementary Material, further inquiries can be directed to the corresponding authors.
