# Supplementary material for: Impact of genetically predicted characterization of mitochondrial DNA quantity and quality on osteoarthritis
Source: Front Genet. 2023 Feb 24;14:1130411. doi: 10.3389/fgene.2023.1130411 (PMC9998702; doi:10.3389/fgene.2023.1130411)
Supplement: Supplementary file 1 [file Table1.DOCX]

Supplementary Material

Impact of genetically predicted characterization of mitochondrial DNA quantity and quality on osteoarthritis

Houpu Liu^1^, Bingyue Cai^1^, Ruicheng Gong^2^, Ye Yang^1^, Jing Wang^1^, Dan Zhou^3,4^, Min Yu^5*^, Yingjun Li^1*^

^1^Department of Epidemiology and Health Statistics, School of Public Health, Hangzhou Medical College, Hangzhou, China

^2^Starbody Clinic, Hangzhou, China

^3^Department of Big Data in Health Science, School of Public Health, Zhejiang University School of Medicine, Hangzhou, China

^4^Vanderbit Genetics Institute, Vanderbilt University Medical Center, Nashville, TN, USA

^5^Zhejiang Provincial Center for Disease Control and Prevention, Hangzhou, China

*** Correspondence:**

Dr Min Yu, Provincial Center for Disease Control and Prevention, 3399 Binsheng Road, 310051, Hangzhou, China. E-mail: [mycdc1234@163.com](mailto:mycdc1234@163.com)

Dr Yingjun Li, Department of Epidemiology and Health Statistics, School of Public Health, Hangzhou Medical College, 481 Binwen Road, 310053, Hangzhou, China. E-mail: 2016034036@hmc.edu.cn

# Supplementary Figures and Tables

## Supplementary Figures

**Supplementary Figure 1**: The forest plot between mitochondria heteroplasmy and different OA phenotypes.


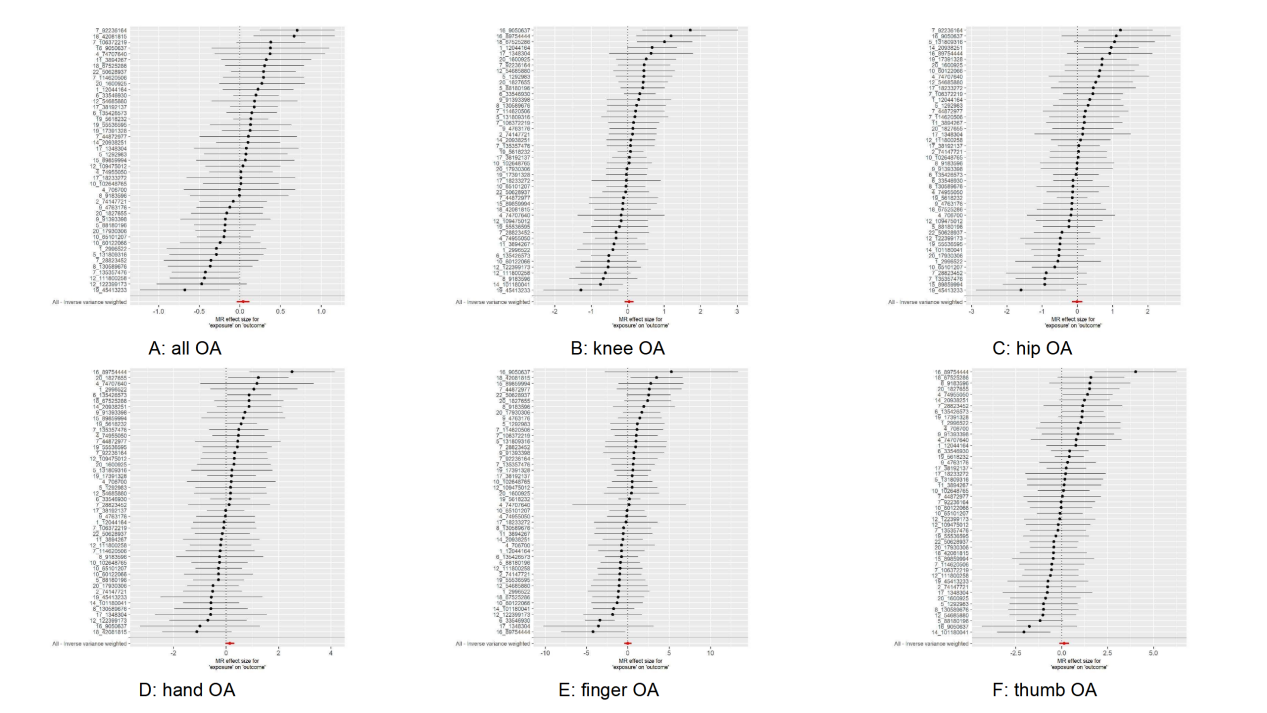


**Supplementary Figure 2**: The leave-one-out plot between mitochondria heteroplasmy and different OA phenotypes.


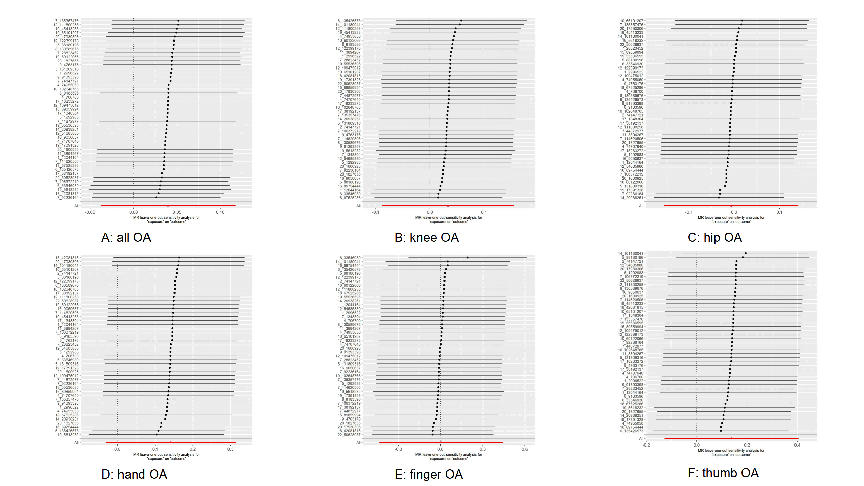


**Supplementary Figure 3**: The scatter plot between mitochondria heteroplasmy and different OA phenotypes.


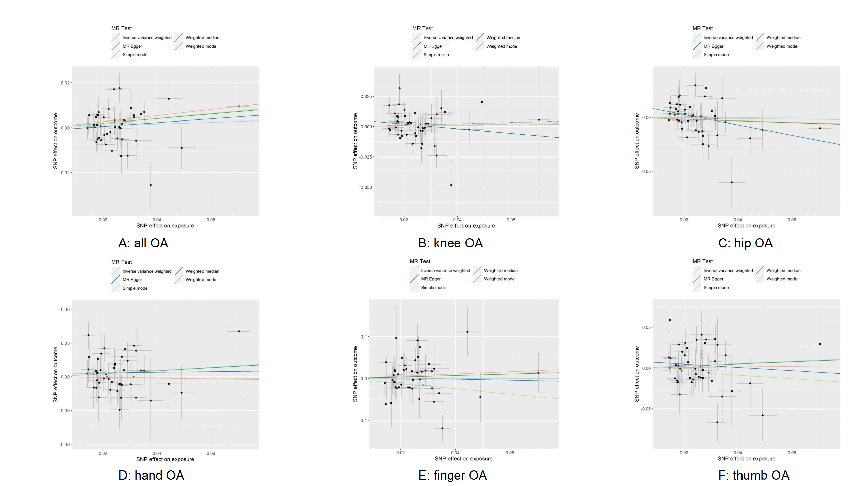


**Supplementary Figure 4**: The funnel plot between mitochondria heteroplasmy and different OA phenotypes.


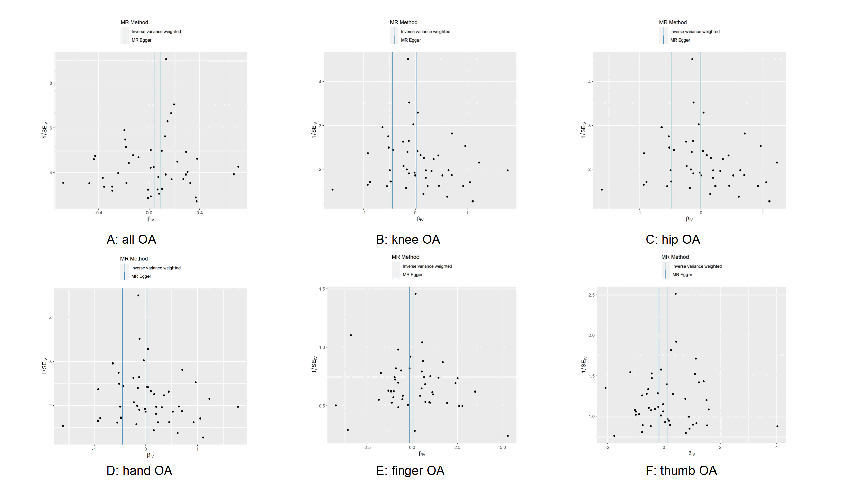


**Supplementary Figure 5**: The forest plot between mtDNA abundance and different OA phenotypes.


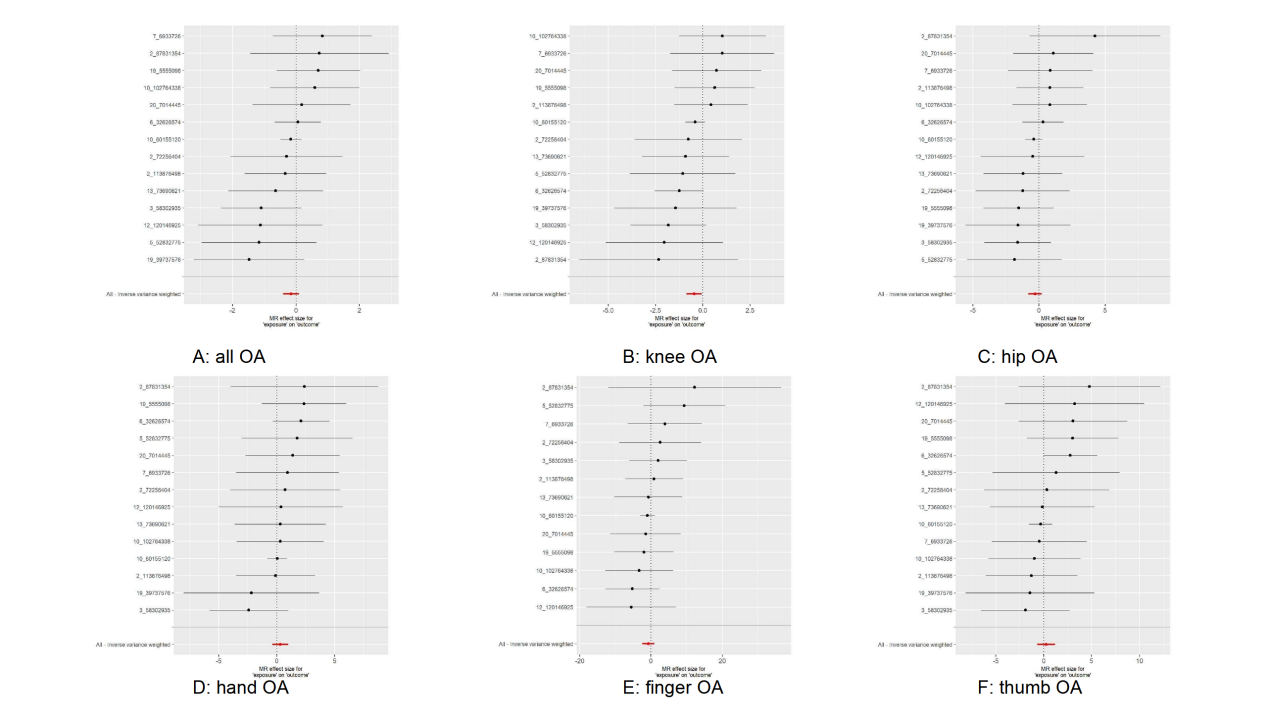


**Supplementary Figure 6**: The leave-one-out plot between mtDNA abundance and different OA phenotypes.


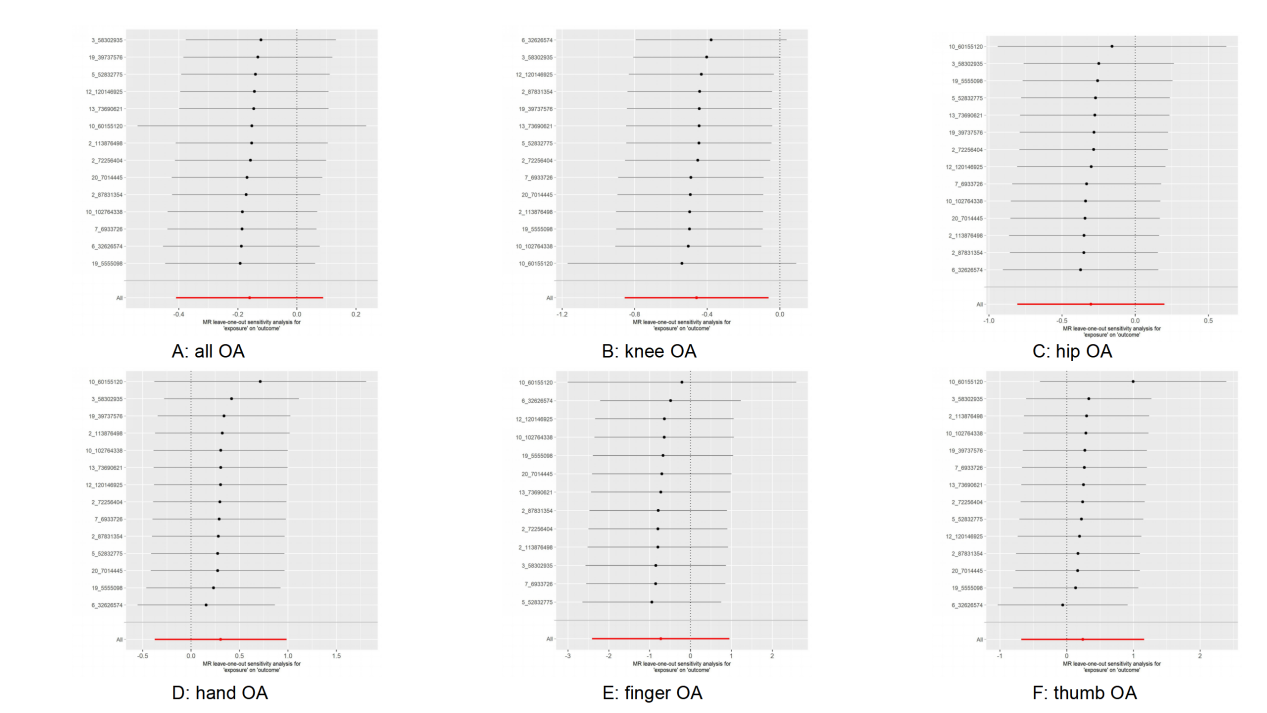


**Supplementary Figure 7**: The scatter plot between mtDNA abundance and different OA phenotypes.


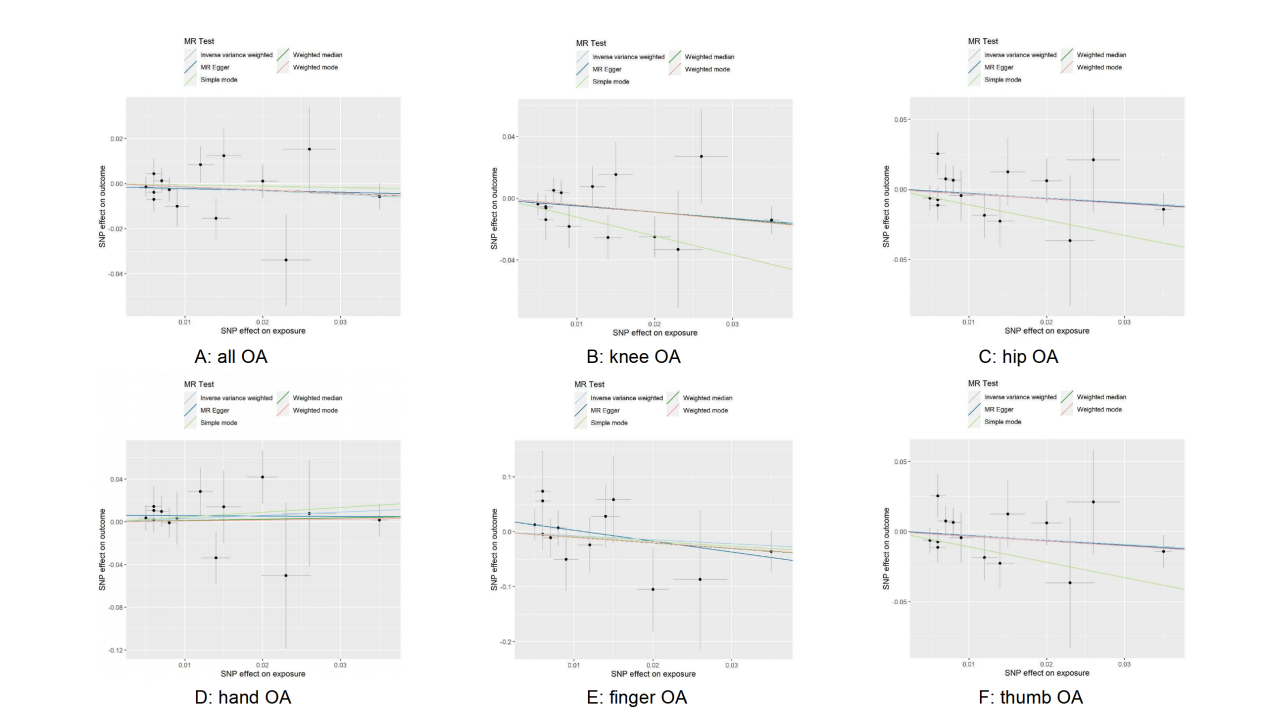


**Supplementary Figure 8**: The funnel plot between mitochondria heteroplasmy and different OA phenotypes.


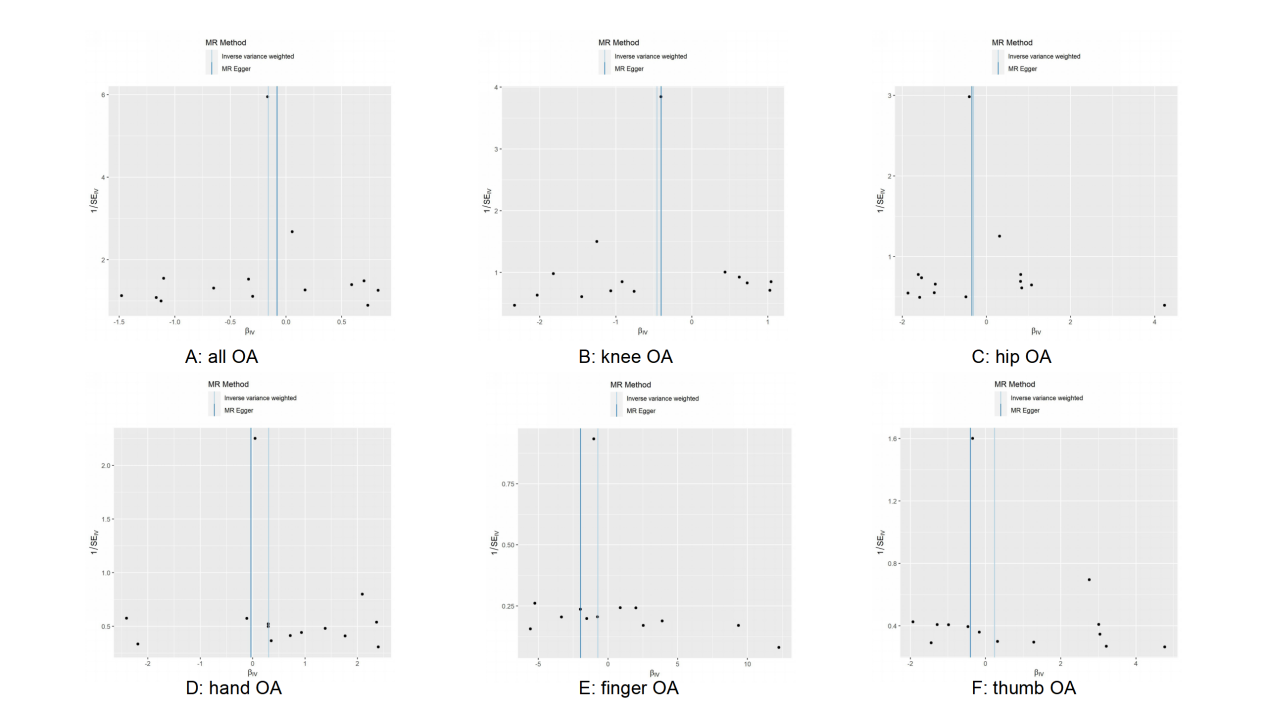


## Supplementary Tables

**Supplementary Table1:** Genetic variants used for Mendelian Randomization analysis of the effect of mitochondrial heteroplasmy on the risk of OA.

| SNP | Chr | Pos | EA | NEA | EAF | Beta | SE | P-value | F statistics |  |
| --- | --- | --- | --- | --- | --- | --- | --- | --- | --- | --- |
| **mitochondrial heterplosmy** |  |  |  |  |  |  |  |  |  |  |
| rs10063311 | 5 | 52832775 | C | G | 0.224 | 0.006 | 0.001 | 9.40E-10 | 1.23E-02 | 36.357 |
| rs1049432 | 10 | 60155120 | G | T | 0.183 | 0.035 | 0.001 | 2.00E-223 | 6.43E-02 |  |
| rs11064881 | 12 | 120146925 | A | G | 0.926 | -0.009 | 0.002 | 1.40E-08 | 1.14E-02 |  |
| rs11679052 | 2 | 72256404 | C | G | 0.578 | -0.005 | 0.001 | 5.00E-08 | 1.10E-02 |  |
| rs12461806 | 19 | 5555098 | A | G | 0.913 | -0.012 | 0.002 | 1.80E-13 | 1.49E-02 |  |
| rs143803034 | 7 | 6933726 | A | G | 0.961 | -0.015 | 0.002 | 1.80E-11 | 1.36E-02 |  |
| rs145232625 | 2 | 87831354 | C | T | 0.257 | -0.006 | 0.001 | 2.00E-09 | 1.21E-02 |  |
| rs2149642 | 20 | 7014445 | C | T | 0.775 | 0.007 | 0.001 | 1.50E-11 | 1.36E-02 |  |
| rs2286639 | 5 | 149579857 | A | G | 0.208 | -0.006 | 0.001 | 1.00E-08 | 1.16E-02 |  |
| rs28539606 | 6 | 32626574 | A | G | 0.147 | 0.02 | 0.002 | 4.00E-23 | 2.00E-02 |  |
| rs370209610 | 19 | 39737576 | C | T | 0.981 | -0.023 | 0.003 | 6.30E-13 | 1.45E-02 |  |
| rs4251979 | 2 | 113876498 | C | T | 0.733 | -0.008 | 0.001 | 1.20E-15 | 1.62E-02 |  |
| rs58678340 | 10 | 102764338 | C | T | 0.014 | -0.026 | 0.003 | 3.20E-14 | 1.53E-02 |  |
| rs73081554 | 3 | 58302935 | C | T | 0.068 | 0.014 | 0.002 | 3.20E-14 | 1.53E-02 |  |
| rs7319964 | 13 | 73690621 | A | T | 0.539 | -0.006 | 0.001 | 7.40E-12 | 1.38E-02 |  |
| F satistics was calculated with adjusted r2 = 0.002069 from the origin GWAS | | | | | | | | | | |

Chr = chromosome; Pos = position; EA = effect allele; NEA = non-effect allele; EAF = effect allele frequency; Beta = effect size estimate; SE = standard error; mean F-statitics across all genetic variants used as instruments for MR analysis with OA as the outcome

**Supplementary Table2:** Genetic variants used for Mendelian Randomization analysis of the effect of mtDNA abundance on the risk of OA.

| SNP | Chr | Pos | EA | NEA | EAF | Beta | SE | P-value | F statistics |  |
| --- | --- | --- | --- | --- | --- | --- | --- | --- | --- | --- |
| **mitochondrial abudance** |  |  |  |  |  |  |  |  |  |  |
| rs4648452 | 1 | 2996522 | C | T | 0.1889 | -0.019 | 0.003 | 5.96E-09 | 93.380 |  |
| rs1474868 | 1 | 12044164 | C | T | 0.4722 | -0.020 | 0.003 | 3.96E-15 |  |  |
| rs831522 | 2 | 74147721 | A | C | 0.3618 | 0.023 | 0.003 | 7.69E-17 |  |  |
| rs35734242 | 4 | 706700 | T | C | 0.4414 | -0.014 | 0.003 | 4.36E-08 |  |  |
| rs12500975 | 4 | 74707640 | T | C | 0.4304 | -0.015 | 0.003 | 1.02E-08 |  |  |
| rs518867 | 4 | 74955050 | C | T | 0.3598 | -0.025 | 0.003 | 1.99E-20 |  |  |
| rs2853672 | 5 | 1292983 | C | A | 0.4871 | -0.018 | 0.003 | 6.55E-12 |  |  |
| rs114694170 | 5 | 88180196 | T | C | 0.05368 | 0.049 | 0.005 | 3.27E-19 |  |  |
| rs2057657 | 5 | 131809316 | A | G | 0.2256 | -0.018 | 0.003 | 3.81E-09 |  |  |
| rs210143 | 6 | 33546930 | C | T | 0.2734 | -0.035 | 0.003 | 3.13E-36 |  |  |
| rs4895441 | 6 | 135426573 | A | G | 0.2734 | 0.031 | 0.003 | 8.51E-28 |  |  |
| rs6976396 | 7 | 28823452 | C | A | 0.1759 | -0.021 | 0.004 | 2.25E-09 |  |  |
| rs12155038 | 7 | 44872977 | G | A | 0.4443 | -0.015 | 0.003 | 1.59E-08 |  |  |
| rs8179 | 7 | 92236164 | C | T | 0.2207 | -0.024 | 0.003 | 2.89E-14 |  |  |
| rs342293 | 7 | 106372219 | C | G | 0.4374 | 0.022 | 0.003 | 1.13E-17 |  |  |
| rs77236693 | 7 | 114620506 | C | T | 0.08151 | 0.030 | 0.004 | 6.21E-12 |  |  |
| rs3110823 | 7 | 135357476 | A | C | 0.1481 | 0.029 | 0.003 | 1.93E-17 |  |  |
| rs4841132 | 8 | 9183596 | G | A | 0.07356 | 0.026 | 0.004 | 2.87E-09 |  |  |
| rs10094039 | 8 | 130589676 | G | A | 0.3718 | -0.017 | 0.003 | 4.06E-11 |  |  |
| rs385893 | 9 | 4763176 | C | T | 0.493 | -0.022 | 0.003 | 7.21E-17 |  |  |
| rs56225686 | 9 | 91393398 | T | A | 0.0507 | -0.032 | 0.005 | 1.64E-09 |  |  |
| rs11006121 | 10 | 60122066 | C | T | 0.3996 | 0.019 | 0.003 | 1.58E-12 |  |  |
| rs10740118 | 10 | 65101207 | G | C | 0.4344 | -0.027 | 0.003 | 4.53E-25 |  |  |
| rs1408343 | 10 | 102648765 | A | G | 0.2336 | -0.026 | 0.003 | 2.41E-19 |  |  |
| rs10835226 | 11 | 3894267 | C | T | 0.3181 | 0.018 | 0.003 | 4.90E-10 |  |  |
| rs35979828 | 12 | 54685880 | C | T | 0.08549 | -0.034 | 0.005 | 1.98E-11 |  |  |
| rs4388979 | 12 | 109475012 | T | G | 0.3767 | 0.019 | 0.003 | 3.20E-13 |  |  |
| rs3809272 | 12 | 111800258 | G | A | 0.3052 | -0.023 | 0.003 | 6.61E-17 |  |  |
| rs11615667 | 12 | 122399173 | C | A | 0.09046 | 0.027 | 0.004 | 4.76E-11 |  |  |
| rs1760940 | 14 | 20938251 | A | C | 0.2455 | 0.028 | 0.003 | 3.55E-21 |  |  |
| rs59488041 | 14 | 101180041 | T | A | 0.1511 | -0.032 | 0.004 | 1.18E-17 |  |  |
| rs3087374 | 15 | 89859994 | C | A | 0.08549 | 0.027 | 0.005 | 1.08E-08 |  |  |
| rs11865642 | 16 | 9050637 | A | C | 0.1829 | -0.018 | 0.003 | 3.06E-08 |  |  |
| rs12924138 | 16 | 89754444 | G | T | 0.3887 | 0.015 | 0.003 | 1.24E-08 |  |  |
| rs2063185 | 17 | 1348304 | C | T | 0.3141 | 0.017 | 0.003 | 6.20E-09 |  |  |
| rs12451555 | 17 | 18233272 | T | G | 0.2445 | -0.017 | 0.003 | 1.98E-08 |  |  |
| rs11078935 | 17 | 38192137 | T | G | 0.3559 | 0.031 | 0.003 | 1.10E-31 |  |  |
| rs16978036 | 18 | 42081815 | G | T | 0.1461 | 0.026 | 0.004 | 1.06E-11 |  |  |
| rs1790961 | 18 | 67525286 | G | T | 0.4761 | -0.018 | 0.003 | 2.10E-12 |  |  |
| rs806709 | 19 | 5618232 | G | A | 0.08549 | 0.070 | 0.004 | 3.03E-62 |  |  |
| rs10419397 | 19 | 17391328 | G | A | 0.2604 | -0.028 | 0.003 | 7.40E-23 |  |  |
| rs1065853 | 19 | 45413233 | G | T | 0.06262 | 0.038 | 0.005 | 1.64E-15 |  |  |
| rs1613662 | 19 | 55536595 | A | G | 0.1451 | 0.024 | 0.003 | 1.96E-12 |  |  |
| rs11696739 | 20 | 1600925 | G | A | 0.3767 | -0.017 | 0.003 | 5.57E-11 |  |  |
| rs156333 | 20 | 1827655 | G | A | 0.4841 | 0.020 | 0.003 | 3.64E-15 |  |  |
| rs11697739 | 20 | 17930306 | C | T | 0.4821 | -0.025 | 0.003 | 5.40E-23 |  |  |
| rs75107793 | 22 | 50628937 | G | A | 0.05865 | 0.044 | 0.005 | 4.66E-19 |  |  |

Chr = chromosome; Pos = position; EA = effect allele; NEA = non-effect allele; EAF = effect allele frequency; Beta = effect size estimate; SE = standard error; mean F-statitics across all genetic variants used as instruments for MR analysis with OA as the outcome.

**Supplementary Table3:** All MR-Steiger directional tests results.

| Exposure | Outcome | The number of SNPs | *p*-value |
| --- | --- | --- | --- |
| Mitochondrial DNA abundance | All OA | 47 | 2.29E-224 |
|  | Hip OA | 47 | 2.68E-57 |
|  | Knee OA | 46 | 4.86E-103 |
|  | Thumb OA | 47 | 1.92E-10 |
|  | Hand OA | 46 | 1.08E-37 |
|  | Finger OA | 46 | 9.58E-13 |
| Mitochondrial heteroplasmy | All OA | 14 | 2.93E-167 |
|  | Hip OA | 14 | 4.29E-56 |
|  | Knee OA | 14 | 9.66E-83 |
|  | Thumb OA | 14 | 1.23E-16 |
|  | Hand OA | 14 | 5.82E-35 |
|  | Finger OA | 13 | 4.64E-17 |
